# Jean Baptiste Octave Landry (1866–1940)

**DOI:** 10.1007/s00415-018-9120-4

**Published:** 2018-11-23

**Authors:** Piotr Skalski, Michał K. Owecki, Anita Maria Magowska

**Affiliations:** Department of History and Philosophy of Medical Sciences, Poznan University of Medical Sciences, Przybyszewskiego 37a, 60365 Poznan, Poland

Jean Baptiste Octave Landry’s greatest achievement was to provide the first description of ascending paralysis, known today as Guillain–Barré syndrome. However, for more than half a century, Landry was eponymous for the disease.

Landry was born on October 10, 1826 in Limoges, France, into a rich family. His father, Michael Landry, was a landowner and came from a wealthy lineage of the French bourgeoisie. Landry’s mother, Catherine-Louis de Théizillat, had Catalonian roots. After completing basic education in Limoges in 1845, Landry began studies at the *Faculté de Médecine de Paris*. His choice of a medical profession was probably inspired by his uncle, Dr de Théizillat, a psychiatrist who headed the mental hospital in Limoges. When a cholera epidemic struck France in 1849, Landry went as a volunteer to the Oise department, north of Paris, to treat its victims. Over 3000 people died in Oise during the epidemic. As it later emerged, the experience gained during this trip influenced Landry’s career and the disease itself was closely associated with his biography. His dedication and devotion to patients made him a well-known and widely respected figure. Soon after Landry returned to Paris, Oise society honored him with a special medal for his achievements in the field of fighting cholera. Thus, began Landry’s scientific career. When only at the beginning of his activity as an *interne des hôpitaux de Paris*, he created his first extensive work: *Mémoire sur le choléra épidémiquede 1849* (Memories of the cholera epidemic of 1849), which was entered for the annual *Concours pour le prix Montyon* (Contest for the Montyon Prize—prize awarded annually by the French Academy of Sciences and the Académie Française), but ultimately did not win.

Landry held his internship in highly reputable Parisian hospitals, among them the Hôtel-Dieu, where he met one of his mentors, Claude-Marie-Stanislas Sandras (1802–1856). In turn, he was supervised by Adolphe-Marie Gubler (1821–1879) at *Hôpital Beaujon*. These two individuals, together with Dr. de Théizillat, made such a strong impression on Landry that he abandoned his initial plans to be a surgeon and continued his internal medicine career. It was during this period that he developed an interest in neurological and pathoneurological issues. In 1852, during his internship with Sandras, he published the work *Recherches physiologiques et pathologiques sur les sensations tactiles* (Physiological and pathological research on tactile sensations), which shed new light on issues of sensory and motor transmission and laid the foundations for knowledge on proprioception.

Despite his young age, Landry was not afraid to criticize more widely recognized researchers dealing with the same subject, such as Pierre-Nicolas Gerdie (1797–1856) and Johannes Peter Müller (1801–1858). In 1854, Landry was deeply affected by his father’s death. In accordance with the social norms of the nineteenth century, he pledged to help his mother maintain two younger siblings. This resulted in unfavorable results in the opening examinations for a position at the local university and hospital. On December 29, 1854, he successfully defended his doctoral thesis, *Considérations générales sur la pathogénie et les indications curatives desmaladies nerveuses* (General considerations of pathogenesis and therapeutic indications of nervous diseases), which consolidated his love for neuroscience [1]. The Chair of the Examination Board was a French physician, Armand Trousseau (1801–1867).

In 1855, Landry extended his Ph.D. thesis and published it under the title *Recherches sur les causes et les indications curatives des maladies nerveuses* (Research into the causes and therapeutic indications of nervous diseases). Forced by financial considerations, shortly after defending his doctoral dissertation he began private practice at *Rue de l’Université* in Paris, which he continued with great success. However, he was still driven by the desire for knowledge and further scientific development. In the same year, 1855, he took up the subject of paralysis and published *Mémoire sur la paralysie du sentiment d’activité musculaire* (On paralysis of muscular activity), in which he discussed loss of muscular activity and sensation [2].

On July 25, 1857, Landry married Claire Giustigniani (1832–1901), a beautiful but impoverished noblewoman. Despite his many professional and family responsibilities, Landry did not abandon his research work. Also in 1857, he published another paper: *De l’emploi du chloroforme et des agents, narcotiques comme agents thérapeutiques et comme moyens de diagnostic dans certaines paralysies* (On the use of chloroform and narcotic agents as therapeutics and diagnostic substances in certain paralysis). In the article, Landry raised the subject of paralysis caused by hysteria and melancholy, indicating the healing influence of chloroform on this form of weakness [3]. He also put forward interesting observations about paralysis. Landry had noticed a group of movement paralyses, unnamed by him, characterized by a symptomatic system, with specific symptoms of: muscle irritability and excitability of nerve trunks, lack of reflex movements, spontaneous convulsive movements, contractures, fibrillary spasms and trembling in current harmed parts of the muscular system.

Landry’s greatest scientific discovery was, however, yet to come. In 1859, he reported one of the first and best, at the time, descriptions of ascending paralysis, in *Gazette Hebdomadaire de Médecine et de Chirurgie* (Weekly Newspaper of Medicine and Surgery) [4]. He described five cases of his own patients, and expanded his study with five other examples known to him from the literature. He distinguished three forms of ascending paralysis: ascending paralysis without sensory symptoms, ascending paralysis with accompanying relief of pain or insensitivity, and a disease that generally progressed with paralysis and sensory symptoms. A classic case of Landry’s ascending paralysis was a 43-year-old paver, with malaise, weakness, fever, pain, and tingling in his toes and hands. Over time, the patient began to lose feeling in his legs and, finally, developed respiratory muscle weakness. The man died in the third week of illness [5]. In the same year, Landry published a work that established his position as an expert in the field of paralysis. In *Traité complet des paralysies* [6] (Complete treaty on paralysis), Landry described contemporary views on the physiology of the brain and medulla. Unfortunately, the planned second volume, which is supposed to relate to the pathophysiology of the nervous system, never appeared, due to Landry’s premature death.

Landry’s reports were widely commented upon, mostly, unfortunately, after his death. In 1892, some of the cases Landry had studied were classified by a Canadian physician Sir William Osler (1849–1919) as acute polyneuritis. In 1896, two American doctors, Pearce Bailey (1865–1922) and James Ewing (1868–1943), described further cases of ascending paralysis, but with particular consideration of anatomopathological changes [7]. In turn, in 1903, a paper devoted to the pathological and bacteriological issues of Landry’s paralysis was published by a British physician, Sir Edward Farquhar Buzzard (1871–1945) [8]. In 1916, French physicians André Strohl (1887–1977), Georges Guillain (1876–1961) and Jean-Alexandre Barré (1880–1967) [9] reported an elevated level of cerebrospinal fluid protein in two French soldiers affected by paralysis and supported their clinical observations by electrophysiological studies. Thus, a new disease eponym was established—*Guillain–Barré syndrome*—and the previously used term *Landry paralysis* was finally abandoned.

Probably because of troublesome migraine headaches and an uncertain financial situation, Landry gave up research and, in October 1859, went to Auteuil, where he took over the management of a hydrotherapeutic sanatorium. The focus at Auteuil was primarily nerve diseases, which led Landry to accept the job. Landry gained considerable wealth in the French province, but also contributed to the development of the sanatorium, making it one of the best centers of this type in France at the time.

In 1865, another cholera epidemic hit France. Once again, Landry went to help the sick, mainly in the area of Auteuil and Boulogne. Unfortunately, he too fell ill after a few days. Realizing the danger of infection, he decided to isolate himself from his family. He died on November 1, 1865, his fellow student, Jean-Martin Charcot (1825–1893), at his side.

Octave Landry is remembered, not only as an excellent doctor-practitioner and researcher, but also as a man of many passions. His father had aroused in him a love of music and Landry was a great singer and violinist. His interests included mountaineering, equestrianism, hunting, and geology. He was also a member of the *Société Médicale d’Observation* and the *Société Anatomique*. Despite his untimely death, Landry’s contributions to the development of neuroscience did not go unnoticed.

## Electronic supplementary material

Below is the link to the electronic supplementary material.

Supplementary material 1 (JPG 6 KB)
