# Supplementary figures and images for: Jean Baptiste Octave Landry (1866–1940)
Source: J Neurol. 2018 Nov 23;266(9):2341–3. doi: 10.1007/s00415-018-9120-4 (PMC6687682; doi:10.1007/s00415-018-9120-4)

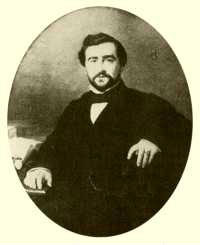

Supplement: Supplementary file 1 — Supplementary material 1 (JPG 6 KB) [file 415_2018_9120_MOESM1_ESM.jpg]
